# A New E-Series Resolvin: RvE4 Stereochemistry and Function in Efferocytosis of Inflammation-Resolution

**DOI:** 10.3389/fimmu.2020.631319

**Published:** 2021-02-10

**Authors:** Stephania Libreros, Ashley E. Shay, Robert Nshimiyimana, David Fichtner, Michael J. Martin, Nicholas Wourms, Charles N. Serhan

**Affiliations:** ^1^ Center for Experimental Therapeutics and Reperfusion Injury, Department of Anesthesiology, Perioperative and Pain Medicine, Brigham and Women’s Hospital and Harvard Medical School, Boston, MA, United States; ^2^ Cayman Chemical, Research and Development Department, Ann Arbor, MI, United States

**Keywords:** lipid mediator, phagocytes, M2 macrophages, pro-resolving mediator, omega-3 fatty acids, pro-resolving, eicosapentaenoic acid

## Abstract

The resolution of the acute inflammatory response is governed by phagocytes actively clearing apoptotic cells and pathogens. Biosynthesis of the specialized pro-resolving mediators (SPMs) is pivotal in the resolution of inflammation *via* their roles in innate immune cells. Resolvin E4 (RvE4: 5S,15S-dihydroxy-eicosapentaenoic acid) is a newly uncovered member of the E-series resolvins biosynthesized from eicosapentaenoic acid (EPA) recently elucidated in physiologic hypoxia. This new resolvin was termed RvE4 given its ability to increase efferocytosis of apoptotic cells by macrophages. Herein, we report on the total organic synthesis of RvE4 confirming its unique structure, complete stereochemistry assignment and function. This synthetic RvE4 matched the physical properties of biogenic RvE4 material, i.e. ultra-violet (UV) absorbance, chromatographic behavior, and tandem mass spectrometry (MS^2^) fragmentation, as well as bioactivity. We confirmed RvE4 potent responses with human M2 macrophage efferocytosis of human apoptotic neutrophils and senescent red blood cells. Together, these results provide direct evidence for the assignment of the complete stereochemistry of RvE4 as 5*S*,15*S*-dihydroxy-6*E*,8*Z*,11*Z*,13E,17*Z*-eicosapentaenoic acid and its bioactions in human phagocyte response.

## Introduction

The acute inflammatory response is a critical and dynamic immunological process during infection and tissue injury (1). Human phagocytes play a pivotal role in host defense and acute inflammation *via* the production of endogenous lipid mediators (LMs), e.g. prostaglandins and leukotrienes, that are potent pro-inflammatory mediators (2). In recent years a novel superfamily of potent endogenous LMs has emerged, coined specialized pro-resolving mediators (SPMs: resolvins, maresins, protectins and lipoxins), which are derived from the essential omega(ω)-3 (docosahexaenoic acid; DHA and eicosapentaenoic acid; EPA) and omega(ω)-6 (arachidonic acid; AA) polyunsaturated fatty acids (PUFAs) (3). SPMs play a fundamental role in the resolution of acute inflammation by governing the temporal and spatial regulation of leukocyte traffic and pro-inflammatory mediators, e.g. eicosanoids, cytokines, and chemokines (3). By definition, each SPM specifically limits neutrophil infiltration and increases the clearance of apoptotic cells by macrophages (3, 4). In this context, macrophages play a central function in the resolution phase of inflammation by actively clearing apoptotic cells (4) and biosynthesizing distinct families of LMs that can be either pro-inflammatory or anti-inflammatory and pro-resolving depending on the macrophage phenotypes and the specific agonists (5, 6). An example of this is with human macrophages: high-mobility group box 1 protein (HMGB1) stimulates the production of pro-inflammatory cytokines and leukotrienes, while high-mobility group box 1 protein together with complement component 1q (C1q) switches macrophages to produce SPMs (7).

EPA (8) is the precursor to the E-series resolvins. EPA’s potential role and use in reducing cardiovascular disease (CVD) has been subject to much debate (9). Recently, using good manufacturing practice production of EPA as a U.S. Food and Drug Administration-approved Icosapent ethyl ester, a human clinical trial reported a substantial reduction in the risk of cardiovascular disease (CVD) (10). While this is a significant advancement for the field of ω-3 based therapeutics, the mechanism of EPA efficacy in reducing CVD remains to be established and its potential role in the resolution of human disease is currently not appreciated. Earlier, we found that hypoxic human vascular endothelial cells released and converted EPA to a novel 18-hydroxyeicosapentaenoic acid (18-HEPE) that is the precursor to resolvin E1 (RvE1) with the complete stereochemistry of 5*S*,12*R*,18*R*-trihydroxy-6*Z*,8*E*,10*E*,14*Z*,16*E*-eicosapentaenoic acid (11, 12). RvE1 is produced *via* transcellular biosynthesis with human neutrophils *via* acetylated cyclooxygenase-2 (COX-2) (12) or microbial cytochrome P450 (13). RvE1 (12) and resolvin E2 (RvE2: 5S,18R-dihydroxy-6*E*,8*Z*,11*Z*,14*Z*,16*E*-eicosapentaenoic acid) (14) both possess potent anti-inflammatory and pro-resolving actions. Resolvin E3 (RvE3: 17R,18R-dihydroxy-5*Z*,8*Z*,11*Z*,13*E*,15*E*-eicosapentaenoic acid) is a vicinal diol that also blocks neutrophil migration (15). The E-series resolvin precursor, 18-HEPE, is also a bioactive mediator with potent actions on cardiovascular tissues (16).

Recently, we encountered and elucidated a new member of the EPA–derived E-series resolvins that we termed Resolvin E4 (RvE4). The stereochemistry of RvE4 was deduced and the double-bond geometry proposed based on the activity of 15-lipoxygenase (15-LOX), namely: 5S,15S-dihydroxy-6*E*,8*Z*,11*Z*,13*E*,17*Z*-eicosapentaenoic acid (see **Illustration Scheme 1**). RvE4 is produced in physiologic hypoxia by human neutrophils and macrophages, and since it stimulates efferocytosis of both senescent erythrocytes (sRBCs) and apoptotic neutrophils, it classifies as a resolving function and with EPA as precursor belongs to the E-series resolvins. Hence, this novel bioactive structure was named RvE4. RvE4 also enhances the resolution of hemorrhagic exudate *in vivo* (17). Recently the biosynthesis of RvE4 was confirmed with purified recombinant human enzymes using 5-lipoxygenase (5-LOX) and 15-LOX with EPA substrate *in vitro* (18). Herein, we establish and confirm the complete stereochemistry of RvE4 and its potent functions in human macrophage efferocytosis.

**Illustration Scheme 1:**
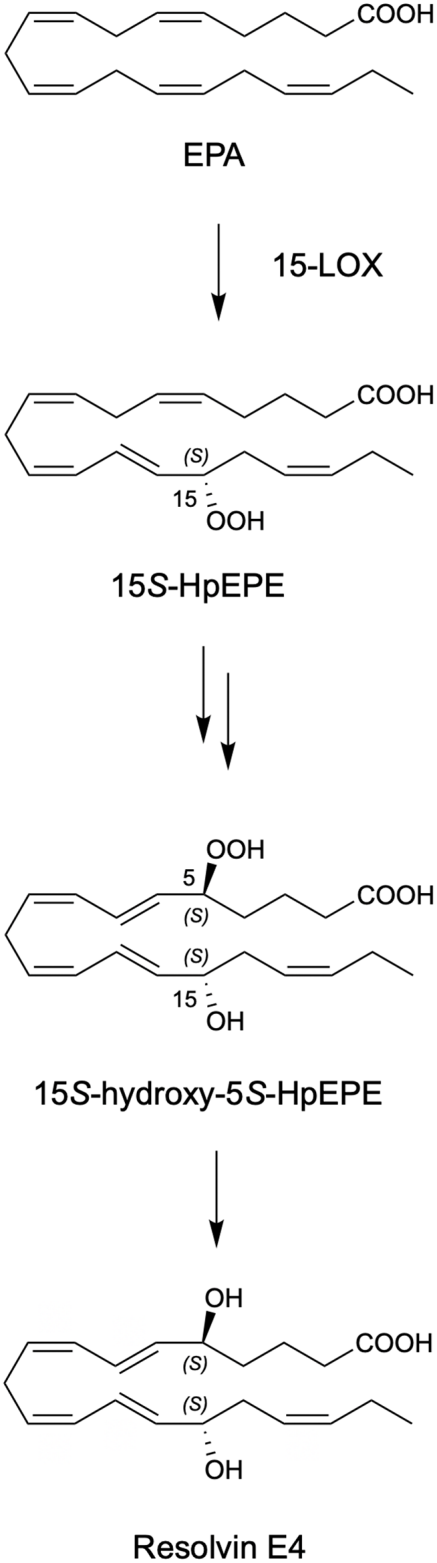
Proposed biosynthesis of Resolvin E4 (RvE4) by hypoxic human leukocytes. The proposed biosynthesis is initiated by lipoxygenation of EPA by 15-lipoxygenase (15-LOX) to 15*S*-hydroperoxy-EPE (15*S*-HpEPE); see (17). This intermediate can be further reduced to the corresponding hydroxyl intermediate 15*S*-HEPE. The resultant products are 15*S*-HpEPE or 15*S*-HEPE and can undergo a second enzymatic lipoxygenation to yield a hydroperoxyl group at the carbon C-5 position, which is reduced to form RvE4.

## Material and Methods

### Synthetic RvE4 Purification

The crude RvE4 was purified using an Agilent 1100 series Prep high-performance liquid chromatography (HPLC) system equipped with a G1315B diode array detector using a Phenomenex Gemini C18 column with 250x21.2 mm dimension, a particle size of 5 micron, and a pore size of 110 angstroms. Resolvin E4 was further purified using methanol (Supelco/Sigma-Aldrich, St. Louis, MO, HPLC grade) and water with 0.1% acetic acid (JT Baker, glacial) mobile phase (68/32 MeOH/water w/0.1% AcOH) at a flow rate of 20 ml/min. The G1315B detector was monitoring 240 nm (bandwidth of 30 nm), 220nm (bandwidth of 30 nm), and 200 nm (bandwidth of 10 nm). The average retention time in these conditions was approximately 15 min.

### Liquid Chromatography-Tandem Mass Spectrometry

The liquid chromatography-tandem mass spectrometry (LC-MS/MS) consists of a 5500 QTRAP mass spectrometer (Sciex, Framingham, MA) equipped with a LC-20AD ultra-fast liquid chromatography (UFLC) system (Shimadzu, Japan). A Poroshell 120 EC-C18 (4.6x100 mm, 2.7 micron, Agilent Technologies, Santa Clara, CA) was kept in a column oven maintained at 50˚C. Resolvin E4 was eluted from this column with a gradient of LC-MS grade methanol (Thermo Fisher, Waltham, MA)/water (Thermo Fisher, Waltham, MA)/acetic acid (Sigma-Aldrich, St. Louis, MO) from 50/50/0.01% *(vol/vol/vol)* to 98/2/0.01% *(vol/vol/vol)* at a flow rate of 0.5 ml/min (19). Targeted multiple reaction monitoring (MRM) for *m/z* 333>115 and enhanced product ion (EPI) mass spectra in negative polarity were used with the following parameters to identify resolvin E4: Collision Energy= −22, Collision Cell Exit Potential= −13, Entrance Potential= −10V, Declustering Potential= −80V, Curtain Gas= 25, Collision Gas= Medium, Ion Spray Voltage= −4,000 V, Temperature= 500°C, Ion Source Gas1 = 60 pounds per square inch (psi), and Ion Source Gas2 = 60 psi. Data were acquired and analyzed with Analyst software version 1.6.2 (Sciex, Framingham, MA) as in (17). Data represented as screen captures from Analyst software.

### Human M2 Macrophages

Human peripheral blood mononuclear cells (PBMC) were obtained from de-identified healthy human volunteers from the Boston Children’s Hospital Blood Bank under protocol # 1999-P-001297 approved by the Partners Human Research Committee. PBMCs were isolated by Ficoll-Histopaque-1077 density-gradient, followed by monocyte purification. Monocytes were then incubated for 7 days in RPMI-1640 medium (Lonza, NJ) containing 10% fetal calf serum, 2 mM L-glutamine, and 2 mM penicillin-streptomycin (Lonza, NJ) at 37°C and differentiated into macrophages through culturing with macrophage colony-stimulating factor (M-CSF) (20 ng/ml) (PeproTech, NJ), followed by polarization for 48 h with IL-4 (20 ng/ml) (PeproTech, NJ) (20, 21).

### Human Neutrophil Purification

Human neutrophils were isolated by dextran sedimentation followed by Ficoll-Histopaque density centrifugation from human peripheral blood (22, 23). Whole blood was obtained from healthy human volunteers giving informed consent under protocol # 1999-P-001297 approved by the Partners Human Research Committee. Apoptotic neutrophils were prepared by plating 1 x 10^7^ cells/ml in 5 ml DPBS (ThermoFisher Scientific, MA) for 24 h in a 100 mm X 20 mm petri dish (Corning, AZ). After 24 h, apoptotic neutrophils were removed from petri dishes with EDTA (5 mM) (Millipore Sigma, MO) and stained with Cell Trace™ Carboxyfluorescein succinimidyl ester (CFSE) at a concentration of 5µM for 30 min at 37°C (ThermoFisher Scientific, MA).

### Human Erythrocyte Isolation

Human peripheral blood red blood cells (RBCs) from healthy human volunteers were isolated by centrifugation and aspiration of the platelet-rich plasma and buffy coat layer. Erythrocytes were purified by resuspension in RPMI-1640 (Lonza, NJ) [10% hematocrit] and centrifugation (500 X g) followed by aspiration of the top 10% of the erythrocyte layer (this purification procedure was carried out with six repetitions) (24). Purified erythrocytes were then resuspended (20% hematocrit) at 4°C for ~24–36 h to induce apoptosis. Erythrocytes were next washed twice with DPBS (ThermoFisher Scientific, MA) and then stained with Cell Trace™ Carboxyfluorescein succinimidyl ester (CFSE) at a concentration of 5mM for 30 min at 37°C (ThermoFisher Scientific, MA).

### Human Efferocytosis of Apoptotic Neutrophils and Senescent Erythrocytes

Human M2 macrophages were seeded in a six-well Corning Costar Flat Bottom Cell culture plate (ThermoFisher Scientific, MA) at a cell density of 2 x 10^6^ cells per well the night before the experiment. M2 macrophages were washed twice with DPBS (ThermoFisher Scientific, MA) to eliminate any cellular debris. Cells were treated with either synthetic RvE4 (10^-11^ M to 10^-7^M), biogenic RvE4 (10^-8^M) or vehicle control (0.01% ethanol vol/vol) 15 min before the addition of CFSE-labeled apoptotic neutrophils [1:3 (M2:apoptotic neutrophils) ratio] or CFSE-labeled senescent erythrocytes [1:50 (M2:sRBC) ratio]. After 60 min of co-incubation at 37°C, plates were washed thoroughly to remove non-ingested cells, and M2 macrophages were fixed with 4% paraformaldehyde for 15 min at 4°C. Cells were washed with DPBS and incubated with 5mM ethylenediaminetetraacetic acid (EDTA) for 15 min before cell scraping. These steps allowed for removal of undigested and membrane bound cells (25).

M2 macrophages were then permeabilized using the BD cytoperm™ permeabilization buffer (BD Biosciences, CA) for 45 min following the manufacturer’s protocol. M2 macrophages were washed twice with BD Perm/Wash buffer (BD Biosciences, CA) and then resuspended in FACS buffer (DPBS, 5% FBS, 2mM EDTA, 2mM NaN_3_). M2 macrophages were then taken to flow cytometry to assess efferocytosis (CFSE intensity). All flow cytometric samples were assessed using BD LSRFortessa (BD Biosciences, CA).

### Software

Flow cytometric data was collected and then analyzed using FlowJo version X (BD Biosciences, CA) as in (17). The chemical structures of E-series resolvins and RvE4 were drawn using ChemDraw Level Professional (Version 16.0.1.4). RvE4 3D spatial filling was done using ChemBioDraw Level Ultra (Version 14.0.0.117) and ChemBio3D Level Ultra (Version 14.0.0.117) (PerkinElmer, Waltham, MA, USA). Graphs were prepared and non-linear regression carried out with Prism software version 8 (GraphPad, CA). Graphic illustrations were created with BioRender software (https://biorender.com/).

### Statistical Analysis

Individual sample incubations were compared by one-way ANOVA with Bonferroni multiple comparisons test or two-tailed student’s t-test. The criterion for statistical significance was p < 0.05.

## Results

### RvE4 Total Organic Synthesis and Stereochemistry

**Figure 1:**
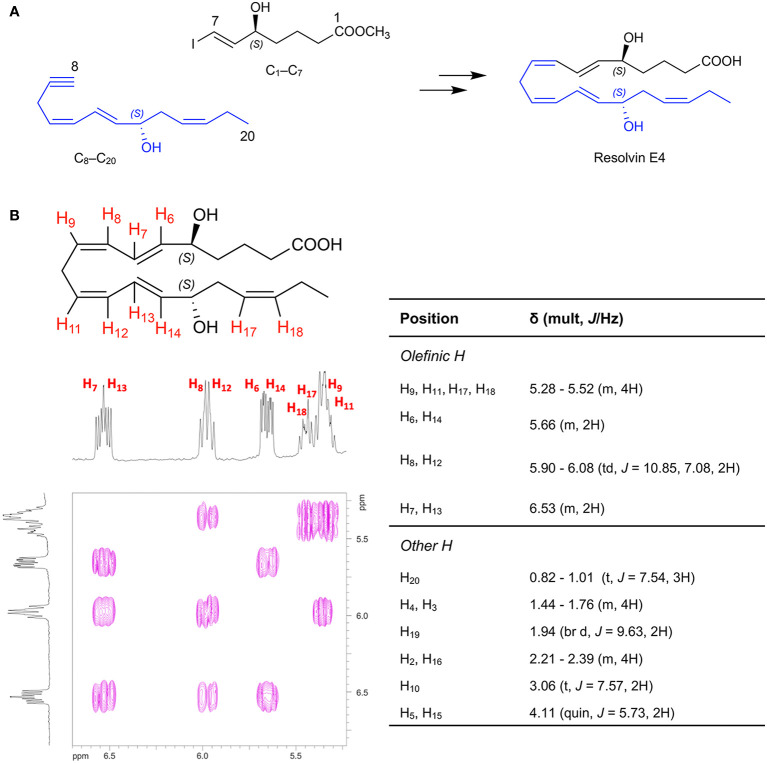
Retrosynthetic strategy for total organic synthesis and spectroscopic properties of Resolvin E4. The synthesis of RvE4 was accomplished by total organic synthesis from stereochemically pure starting building blocks and was characterized using NMR spectroscopy. **(A)** Synthetic precursors were prepared from enantiomerically pure commercially available starting materials and assembled using carbon-carbon bond coupling reactions between precursors C1–C7 (black), and C8–C20 (blue) to ensure absolute regio- and stereochemical control. **(B)** Double-bond geometry was assigned using two-dimensional ^1^H-^1^H NMR using a JNM ECZ-400S NMR spectrometer with a 400MHz magnet at 20.7°C on a JEOL ROYALPROBE^TM^ and referenced to the methanol-*d_4_* (CD_3_OD) internal standard. The purple plot depicts positive contours of cross-peaks along the diagonal axis allowing for the full and detailed proton assignment. The enlarged region highlights the Z/E olefinic protons H_6_-H_9_, H_11_-H_14_, and H_17_-H_18_. This, in addition to the full ^1^H-NMR spectrum of chemical shifts and coupling constants, confirmed the structure of Resolvin E4.

To assign the complete stereochemistry of RvE4 and determine whether synthetic RvE4 shares reported physical and biological functions (17), it was critical to establish the spectroscopic and physical properties of the new synthetic RvE4. The structure and stereochemistry of synthetic RvE4 were precise on the basis of its total organic synthesis from chiral starting materials of known stereochemistry as well as stereocontrolled chemical reactions as outlined in **Figure 1A**. Synthetic RvE4 was prepared to target RvE4 structure deduced earlier using the isolated well studied 15-lipoxygenase (LOX) enzyme that inserts molecular oxygen into 1,4-cis pentadiene predominantly in the *S* configuration (17). The total synthesis was accomplished in 21 steps to afford stereochemically pure 5*S*,15*S*-dihydroxy-6*E*,8*Z*,11*Z*,13*E*,17*Z*-eicosapentaenoic acid, RvE4. The synthesis began with defined building blocks of known stereochemistry to furnish the carbons C1-C7 fragment (**Figure 1A**, black). Next, the carbons C8-C20 fragment (**Figure 1A**, blue) was generated from chirally pure (*R*)-(+)-glycidol to unambiguously install the carbon C-15 position *S* alcohol chirality. With both the carbon C1–C7 and carbon C8–C20 key intermediates at hand, these fragments were joined together using a carbon-carbon cross-coupling reaction to form the eicosapentaenoate carbon backbone of RvE4 methyl ester.

The methyl ester was further hydrolyzed to afford crude RvE4 (**Figure 1A**). The product was isolated as in **Figure 1A** using HPLC [Gemini C18, 5 micron, 250 x 21.2 mm, 110Å, monitored at 240 nm (bandwidth of 30 nm), 220nm (bandwidth of 30 nm), and 200 nm (bandwidth of 10 nm), methanol/water/acetic acid (AcOH) 68/32/0.1%, 20 ml/min]. Further evidence for structural verification was obtained for this product from its full ^1^H nuclear magnetic resonance (NMR) spectroscopy, and the *Z*/*E* configuration of the double bonds was determined and confirmed using 2D ^1^H NMR (COSY) as shown in **Figure 1B** and **Supplementary Figure 1A**.

### RvE4 Physical Properties: Matching of Organic Synthetic to Biogenic Material

**Figure 2:**
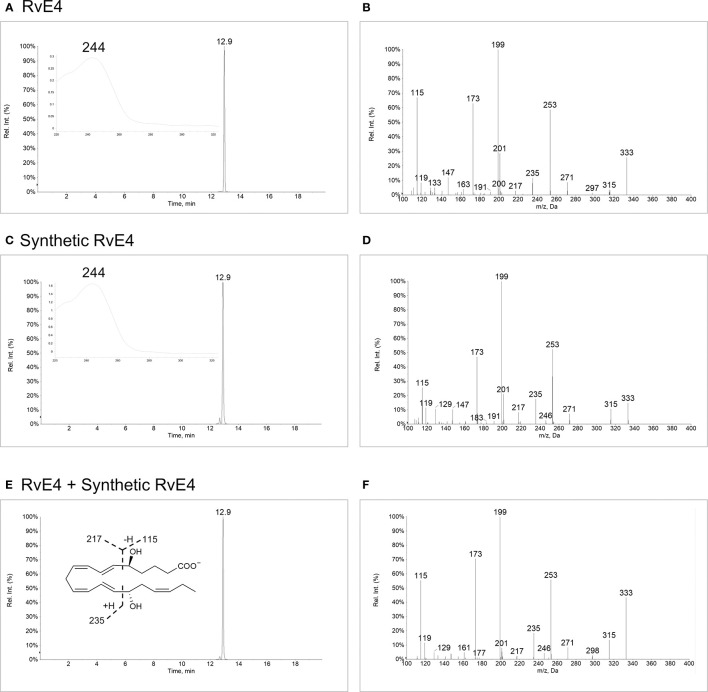
Matching physical properties of biogenic and synthetic RvE4. Screen captures of **(A)** Biogenic RvE4 monitored by liquid chromatography-tandem mass spectrometry (LC-MS/MS) in negative ion MRM mode for *m/z* 333>115. *Inset:* UV spectrum with ${\lambda_{\text{max}}}^{\text{MeOH}}=244\;nm$. **(B)** MS/MS fragmentation spectrum with ions matching RvE4. **(C)** Synthetic RvE4 monitored by LC-MS/MS in negative ion MRM mode for *m/z* 333>115. *Inset:* UV spectrum with ${\lambda_{\text{max}}}^{\text{MeOH}}=244\;nm$. **(D)** MS/MS fragmentation spectrum with ions matching RvE4. **(E)** Biogenic and synthetic RvE4 were co-injected and monitored by LC-MS/MS in negative ion MRM mode for *m/z* 333>115. *Inset:* RvE4 structure with proposed fragmentations. **(F)** MS/MS fragmentation spectrum with ions matching RvE4. Arrows on y-axes indicate threshold; 5% for chromatograms **(A, C, E)**, 1% for spectrum **(B, D, F)**.

To determine whether synthetic RvE4 shares the identical physical and biological properties of biogenic RvE4, it was essential to establish the chromatographic behavior, ultra-violet (UV) absorbance, and structural composition of both molecules. Biogenic RvE4 was prepared by incubating EPA with soybean 15-LOX and purified by high-performance liquid chromatography with a chiral column (HPLC) (17). This biogenic preparation of RvE4 matched endogenously biosynthesized RvE4 by human M2 macrophage-neutrophil co-incubations (17). The ultraviolet (UV)-chromophore of the biogenic RvE4 showed a single broad absorption band at ${\lambda_{\text{max}}}^{\text{MeOH}}=244\;nm$ (solvent, methanol) (**Figure 2A**, inset), which is characteristic of two separate conjugated diene double bond systems in the RvE4 carbon backbone. This UV absorbance max in methanol was in accordance with our earlier published results obtained from M2 macrophage-neutrophil co-incubations, *i.e.* ${\lambda_{\text{max}}}^{\text{MeOH}}=244\;nm$, that produced RvE4 from endogenous stores of EPA (17). We next assessed the chromatographic behavior of biogenic RvE4 using reverse-phase ultra-fast liquid chromatography. Biogenic RvE4 eluted at 12.9 min as a single and major peak (**Figure 2A**). Tandem mass spectrometry analysis (MS/MS screen capture) of biogenic RvE4 revealed a fragmentation pattern with a parent ion of *m/z* 333=M-H and daughter ions of *m/z* 315=M-H-H_2_O, *m/z* 271=M-H-H_2_O-CO_2_, *m/z* 253=M-H-2H_2_O-CO_2_, *m/z* 217 = 235-H_2_O, *m/z* 199 = 217-H_2_O, and *m/z* 173 = 235-H_2_O-CO_2_ as shown in **Figure 2B**.

Synthetic RvE4 demonstrated an essentially identical UV absorbance of ${\lambda_{\text{max}}}^{\text{MeOH}}=244\;\mathrm{nm}$; see **Figure 2C**, inset, as well as the chromatographic behavior by obtaining an identical retention time of 12.9 min (**Figure 2C**) as biogenic RvE4. MS/MS direct screen capture analysis of synthetic RvE4 gave essentially the same fragmentation pattern as biogenic RvE4, i.e. with a parent ion of *m/z* 333=M-H and daughter ions of *m/z* 315=M-H-H_2_O, *m/z* 271=M-H-H_2_O-CO_2_, *m/z* 253=M-H-2H_2_O-CO_2_, *m/z* 217 = 235-H_2_O, *m/z* 199 = 217-H_2_O, and *m/z* 173 = 235-H_2_O-CO_2_ (**Figure 2D**). This fragmentation pattern matches that of reported endogenous RvE4 isolated from human and murine leukocytes (17). Having matched these physical properties of biogenic RvE4 to those of the synthetic RvE4, we next sought evidence to determine if 50% co-injections of both compounds have identical physical properties in these same conditions. Co-injection of biogenic RvE4 with the synthetic RvE4 confirmed their identical chromatographic behavior, which co-eluted at 12.9 min beneath a single peak (**Figure 2E**). MS/MS direct screen capture analysis revealed that co-injection material has an identical fragmentation pattern (**Figures 2B, D**) as with parent ion of *m/z* 333=M-H and daughter ions of *m/z* 315=M-H-H_2_O, *m/z* 271=M-H-H_2_O-CO_2_, *m/z* 253=M-H-2H_2_O-CO_2_, *m/z* 217 = 235-H_2_O, *m/z* 199 = 217-H_2_O, and *m/z* 173 = 235-H_2_O-CO_2_ (**Figure 2F**). RvE4 structure with major fragmentation points (*m/z* 115, *m/z* 217, and *m/z* 235) is shown in **Figure 2E**, inset. The present results with both biogenic and synthetic RvE4 confirmed the original reported physical properties of RvE4 and further verified its unique stereochemistry (see **Supplementary Figure 1**).

### RvE4 Increases Efferocytosis in Human M2 Macrophages

**Figure 3:**
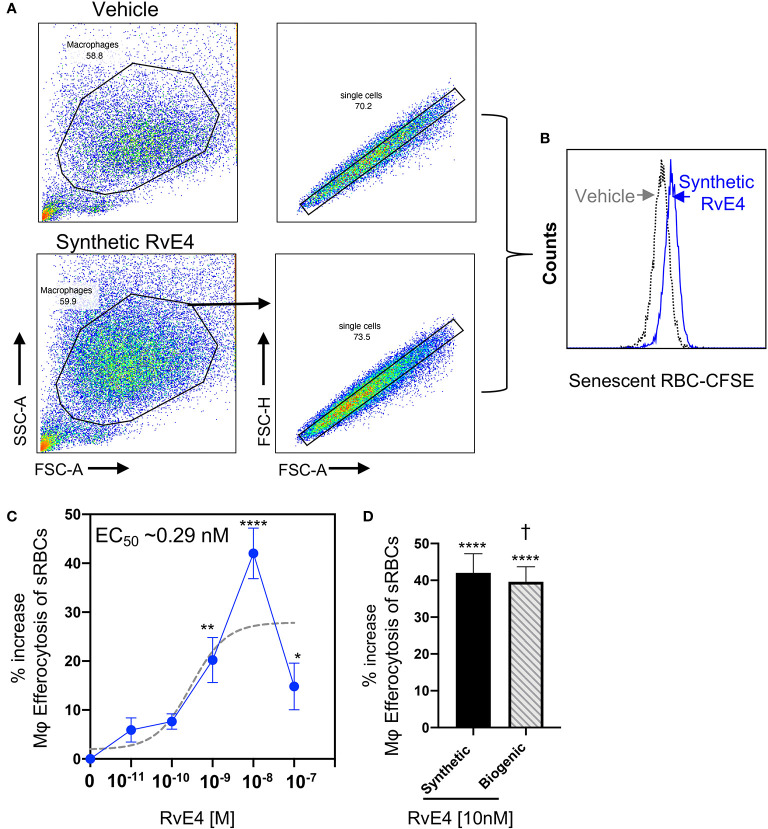
RvE4 increases human M2 macrophage efferocytosis of senescent red blood cells. Human M2 macrophages were plated on a 6-well plate (2 x 10^6^ cells/well), incubated with synthetic RvE4 (10^-11^M to 10^-7^M), biogenic RvE4 (10^-7^ M), or vehicle (0.01% ethanol *vol/vol*) for 15 min followed by CFSE-labeled senescent red blood cells (sRBCs) (1:50, M2:sRBCs). After 60 min of co-incubation at 37°C, plates were washed thoroughly to remove non-ingested sRBCs, and cells were fixed with DPBS containing 4% paraformaldehyde for 15 min on ice. Cells were washed with DPBS and removed from plates with a cell scraper in DPBS. M2 macrophages were then taken to flow cytometry to assess efferocytosis. **(A)** Flow cytometry gating strategy for M2 macrophages. **(B)** Representative histogram of intracellular CFSE-labeled sRBCs. **(C)** Dose-Response: percent increase in M2 efferocytosis of sRBCs above vehicle by synthetic RvE4 (solid blue line). EC_50_ was estimated using non-linear regression (dashed gray line) with log (agonist) vs. response (three parameters). **(D)** Comparison between synthetic RvE4 (10 nM) and biogenic RvE4 (10 nM): percent increase of M2 efferocytosis of sRBCs above the vehicle **(C, D)**. Results are the percent of efferocytosis above vehicle and expressed as mean ± SEM, n=5 (sRBCs). *p < 0.05, **p < 0.01, and ****p < 0.0001 when compared to vehicle (as control). † (p=0.73) no statistically significant differences when compared between synthetic and biogenic RvE4. Statistical analysis was carried out using one-way ANOVA with Bonferroni multiple comparisons test **(C)** or two-tailed Student’s t-test **(D)**.

Macrophage efferocytosis is one of the hallmarks of the resolution of inflammation, and pro-resolving mediators are key bioactive regulators of efferocytosis (3, 4). RvE4 is a potent stimulator of macrophage efferocytosis of senescent red blood cells (sRBCs) (17). Macrophage erythrophagocytosis is an essential mechanism for the removal of sRBCs in the spleen as this organ is the largest filter of RBCs in the human body (26). Therefore, we next determined whether the synthetic RvE4 enhances macrophage efferocytosis of sRBCs. For this experiment, human M2 macrophages were incubated with synthetic RvE4 (0–100 nM) or vehicle control (0.01% ethanol *vol/vol)* prior to exposure with sRBCs to assess the direct actions on human M2 efferocytosis by flow cytometry. Representative flow cytometry gating strategy of M2 macrophages and histograms of intracellular CFSE-labeled sRBCs from vehicle control and synthetic RvE4 are reported in **Figures 3A, B**. Synthetic RvE4 increased macrophage efferocytosis of sRBCs in a concentration-dependent fashion with the highest statistically significant increase at 1nM and 10nM (**Figure 3C**) compared to vehicle, with an estimated EC_50_ of ~0.29 nM for synthetic RvE4. Since M2 macrophages treated with 10 nM of synthetic RvE4 had the highest increase in efferocytosis of sRBCs, we next examined if biogenic RvE4 at 10 nM had the same ability to stimulate efferocytosis in human M2 macrophages. Both synthetic RvE4 and biogenic RvE4 potently increased M2 efferocytosis of sRBCs by as much as ~40% at 10nM concentration (**Figure 3D**). There were no statistically significant differences noted between synthetic and biogenic RvE4 (p=0.73) by analysis of variance, indicating that both mediators gave an identical biological response. These results demonstrate that synthetic and biogenic RvE4 have essentially the same potencies in stimulating human macrophage efferocytosis of sRBCs.

**Figure 4:**
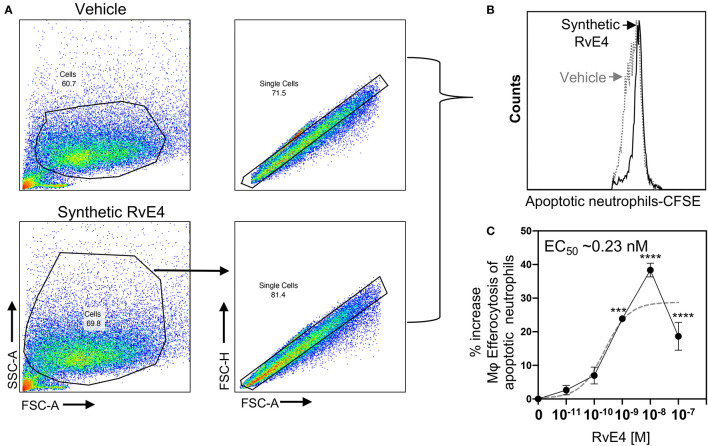
Synthetic RvE4 increases human M2 macrophage efferocytosis of apoptotic human neutrophils. Human M2 macrophages were plated on a 6-well plate (2 x 10^6^ cells/well), incubated with synthetic RvE4 (10^-11^ to 10^-7^M) or vehicle (0.01% ethanol) for 15 min followed by CFSE-labeled apoptotic neutrophils (1:5 M2: apoptotic neutrophils). After 60 min of co-incubation at 37°C, plates were washed thoroughly to remove non-ingested neutrophils. M2 macrophages were fixed with DPBS containing 4% paraformaldehyde for 15 min on ice. Cells were washed with DPBS and removed from plates with a cell scraper in DPBS. Cells were then taken to flow cytometry to assess efferocytosis. **(A)** Flow cytometry gating strategy for M2 macrophages. **(B)** Representative histogram of intracellular CFSE-labeled apoptotic neutrophils. **(C)** Dose-Response: percent increases in M2 macrophage efferocytosis of apoptotic PMN above vehicle by synthetic RvE4 (solid black line). EC_50_ was estimated using non-linear regression (dashed gray line) with log (agonist) vs. response (three parameters). Results are represented as mean ± SEM. n=3 healthy human donors. ***p < 0.001 and ****p < 0.0001 compared to vehicle (as control). Statistical analysis was carried out using one-way ANOVA with Bonferroni multiple comparisons test.

Neutrophils are often the first leukocytes to infiltrate an inflammatory site in large numbers and therefore their effective elimination is a prerequisite for the resolution of inflammation (3, 4, 27, 28). SPMs, including RvE4, enhance macrophage efferocytosis of apoptotic neutrophils *in vitro* and *in vivo* with both human cells and murine models of inflammation (3, 17). Given that RvE4 enhanced efferocytosis of sRBCs, we set out to determine if synthetic RvE4 enhances M2 macrophage efferocytosis of human apoptotic neutrophils. Human M2 macrophages were incubated with synthetic RvE4 (0–100 nM) or vehicle control (0.01% ethanol *vol/vol*) prior to exposure with CFSE-labeled apoptotic human neutrophils, and efferocytosis was assessed using flow cytometry. Representative flow cytometry gating strategy of M2 macrophages and histograms of intracellular CFSE-labeled apoptotic neutrophils from vehicle control and synthetic RvE4 are shown in **Figures 4A, B**. Synthetic RvE4 increased macrophage efferocytosis of apoptotic human neutrophils in a concentration-dependent fashion, with the highest statistically significant increase at 1nM and 10nM (**Figure 4C**) compared to vehicle control, with an estimated EC_50_ of ~0.23nM for synthetic RvE4. The increase of ~39% of M2 efferocytosis by the synthetic RvE4 at 10nM is in accordance with the published results of M2 macrophage efferocytosis of apoptotic neutrophils by the biogenic RvE4 (17). Taken together, these results demonstrate the potent biological actions of synthetic RvE4 in enhancing efferocytosis with human M2 macrophages.

## Discussion

In the present report, we matched both the physical properties of RvE4 and its potent functions with human M2 macrophages to material produced *via* total organic synthesis permitting us to assign and confirm the proposed stereochemistry of RvE4 (17). Through matching studies using LC-MS/MS (co-elution and fragmentation), UV spectrum absorbance and biological actions, we have assigned the complete double-bond geometry of RvE4 as 5*S*,15*S*-dihydroxy-6*E*,8*Z*,11*Z*,13*E*,17*Z*-eicosapentaenoic acid (**Figures 1** and **2**). Therefore, synthetic RvE4 can be used as both a standard for LC-MS/MS-based mediator lipidomics as well as for further investigations to define potentially additional RvE4 functions in diverse biological systems.

**Figure 5:**
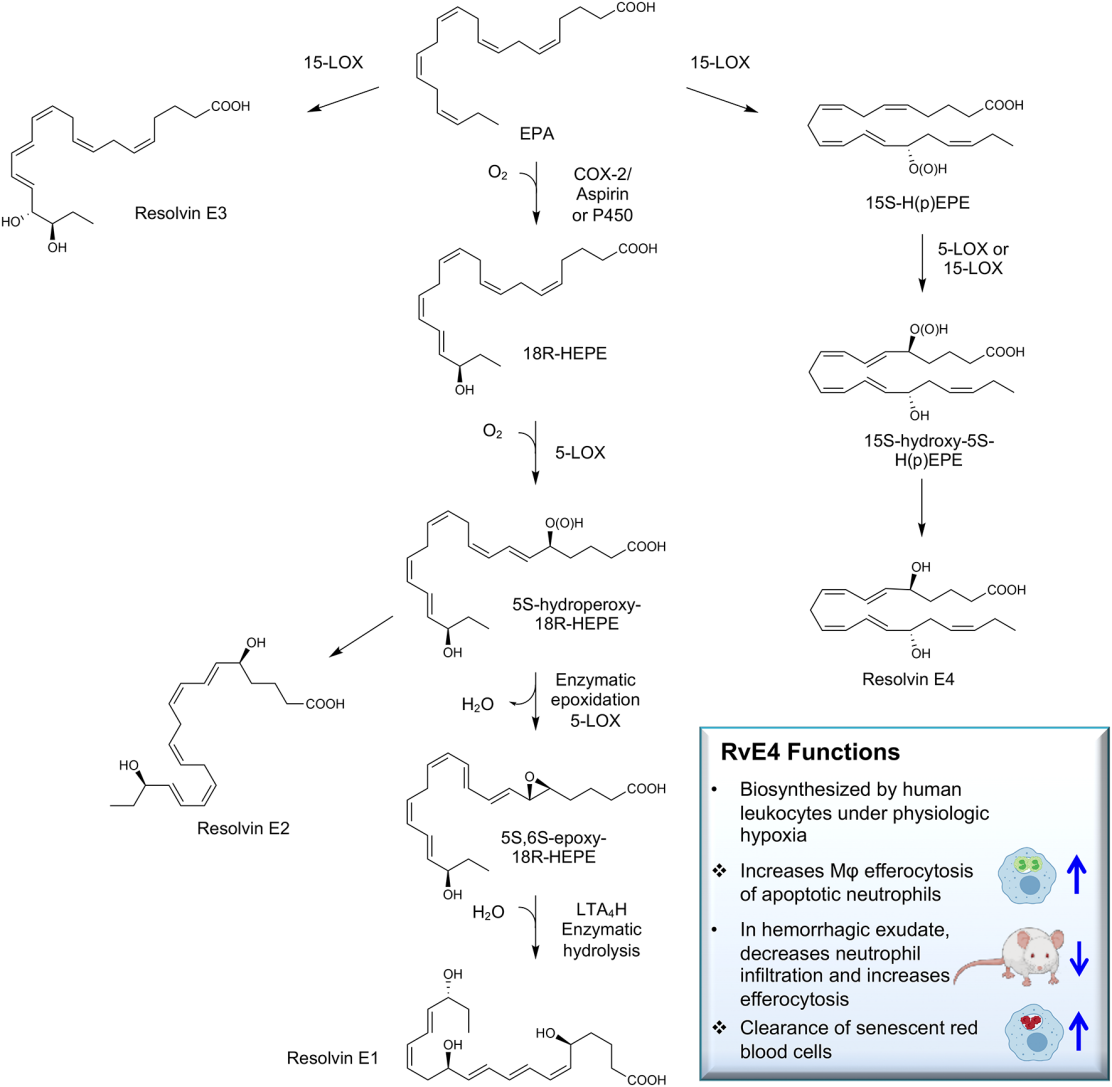
Biosynthetic scheme for Resolvin E4 and E-series resolvins. Eicosapentaenoic acid (EPA) is converted to 18*R*-HEPE *via* acetylation of COX-2 by aspirin (12) or P450 (13), which is subsequently lipoxygenated by 5-LOX to produce the intermediate 5S-hydroperoxy-18*R*-HEPE. 5S-hydroperoxy-18*R*-HEPE can be either reduced to RvE2 (5*S*,18*R*-dihydroxy-6*E*,8*Z*,11*Z*,14*Z*,16*E*-eicosapentaenoic acid) (14) or to 5*S*,6*S*-epoxy-18*R*-HEPE by enzymatic epoxidation by 5-LOX confirmed by trapping experiments. 5S,6S-epoxy-18*R*-HEPE can be further hydrolyzed to produce RvE1 (5*S*,12*R*,18*R*-trihydroxy-6*Z*,8*E*,10*E*,14*Z*,16*E*-eicosapentaenoic acid) (11, 12). EPA can also be lipoxygenated by 15-LOX to produce RvE3 (17*R*,18*R*-dihydroxy-5*Z*,8*Z*,11*Z*,13*E*,15*E*-eicosapentaenoic acid) (15). Stereochemistry and proposed biosynthetic route of RvE4 and confirmed actions (❖ as demonstrated in the present paper) adding to the family of E-series resolvins derived from EPA.

RvE4 was uncovered from physiological hypoxic biosynthesis of EPA, *via* lipoxygenation, by human macrophages and neutrophils (17). M2 macrophages play critical roles in the resolution of inflammation by virtue of their capacity to carry out efferocytosis (3, 4), wound repair (3, 29), and production of SPMs (5). In the present experiments, RvE4 proved to be a potent agonist of M2 macrophage efferocytosis of neutrophils, giving an estimated EC_50_ ~0.23nM (**Figure 4**) and an EC_50_ ~0.29nM for the efferocytosis of sRBCs (**Figure 3**). In human macrophages and neutrophils, RvE4 biosynthesis is dependent on the substrate availability of EPA released from both phospholipases (PL) and triglycerides (17). EPA is converted by 15-LOX to 15S-HpEPE, which becomes a substrate for further lipoxygenation by either 5-LOX or a second enzymatic turn of 15-LOX to produce 15S-hydroxy-5S-HpETE; this is further reduced to RvE4 (**Figure 5**). This route of RvE4 biosynthesis by human phagocytes was recently confirmed by Kutzner et al. using recombinant human 5- and 15-LOX co-incubations *in vitro* (18). Recently, EPA was shown to be converted by wild type and recombinant engineered lipoxygenases to double dioxygenation products 5*S*,12*S*-diHEPE and RvE4 on large-scale studies (30). In addition, it is possible that the lipoxygenation of EPA is initiated *via* hydrogen abstraction at carbon position C7 by 5-LOX (**Figure 5**) to biosynthesize RvE4 in human neutrophils or by transcellular biosynthesis (31). Since earlier results demonstrate the conversion of EPA to 15*R*-HEPE *via* acetylated COX-2 (12, 13), it is plausible that lipoxygenation by 5-LOX and further conversion to the corresponding alcohol could give rise to 15*R*-RvE4. This 15*R*-RvE4 could be biosynthesized either from the acetylation of COX-2 by acetyl-CoA and sphingosine *via* sphingosine kinase 1 (SphK1) (32) or modified by S-nitrosylation by inducible nitric oxide synthase (33). Also, since 5-LOX can be phosphorylated to produce 15*R*-HETE and further converted to 15*R*-lipoxin A_4_ (LXA_4_) (34–36), it is likely that with EPA as a substitute phosphorylated 5-LOX can produce 15R-HEPE and subsequently be converted to 15*R*-RvE4.Therefore RvE4 can be biosynthesized from EPA potentially *via* multiple biosynthetic routes and now can be included in the E-series of pro-resolving mediators; see **Table 1**. The 15-LOX initiated route of RvE4 biosynthesis simplifies the production and biosynthesis of this mediator that can occur in the absence of enzyme modification (i.e. phosphorylation, acetylation, nitrosylation of COX-2) or aspirin therapy.

**Table 1:** Eicosapentaenoic acid (EPA) derived specialized pro-resolving mediators (SPMs) and stable analogs: actions and human production.

Full name/Abbreviation	Chemical name****	Structure****	Pro-resolving actions****	Organ protection****	Quantities in humans****
**Resolvin E1** (RvE1)	5*S*,12*R*,18*R*-trihydroxy-6*Z*,8*E*,10*E*,14*Z*,16*E*-eicosapentaenoic acid	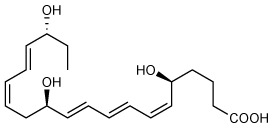	Stops PMN (12, 14, 37–39) and dendritic cell migration (11, 40). Reduces pro-inflammatory cytokines (41). Enhances macrophage phagocytosis & efferocytosis (37, 38). Enhances bacterial clearance (41). Inhibits TRP channels (42). Modulates T cell responses (42). Regeneration of periodontal ligament stem cells (43). Inhibits platelet aggregation (44).	Peritonitis (11, 12, 14, 37, 38), air pouch (12), CLP/sepsis (45), bacterial (41), I/R injury (46), diabetes (47), obesity (48), colitis (39, 49, 50), lung inflammation (51), kidney injury (52), depression (53, 54), atherosclerosis (55) (56), bone (57), reduces tumor growth (58), Dermatitis (59), *Candida albicans* (60), pain (61), and herpes (62).	Plasma 3.9–4.5 pg/ml (63) Plasma of Arthritis 96.1 pg/ml (64) Arthritis Synovial fluid 24.6 pg/ml (64) Plasma of Type 2 diabetes mellitus 45.9–50 pg/ml (65) Plasma of Peripheral artery disease (OMEGA-PAD II trial) 0.32–0.62 pg/ml (66) Cord Blood 34.1 pg/ml (67) Human Breast milk 8.8 pg/ml (68) Blister 0.1–0.3 pg/blister (69) Metabolic syndrome (weight loss program) 378-1339 pg/4.5 × 10^6^ PMN (70)
**18S-Resolvin E1** (18S-RvE1)	5*S*,12*R*,18*S*-trihydroxy-6*Z*,8*E*,10*E*,14*Z*,16*E*-eicosapentaenoic acid	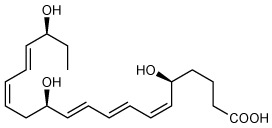	Stops PMN migration, reduces pro-inflammatory cytokines, and enhances macrophage phagocytosis & efferocytosis (38).	Peritonitis (38).	
**18-oxo-Resolvin E1** (18-oxo-RvE1)	5*S*,12*R*-dihydroxy 6*Z*,8*E*,10*E*,14*Z*,16*E*-18-oxo-eicosapentaenoic acid	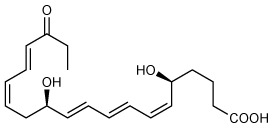	Stops PMN migration, reduces pro-inflammatory cytokines (71).	Peritonitis (71).	
**Resolvin E2** (RvE2)	5*S*,18*R*-dihydroxy-6*E*,8*Z*,11*Z*,14*Z*,16*E*-eicosapentaenoic acid	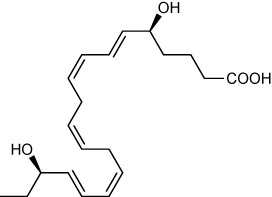	Stops PMN migration (14, 38). Down-regulates leukocyte integrins (72).	Peritonitis (14, 38), and depression (53).	Plasma 2.3–2.7 pg/ml (63) Plasma of Arthritis 68.8 pg/ml (64) Arthritis Synovial fluid 774.2 pg/ml (64) Plasma of Type 2 diabetes mellitus 59.7–82.3 pg/ml (65) Cord Blood 39.0 pg/ml (67) Plasma of Peripheral artery disease (OMEGA-PAD II trial) 9.7–13 pg/ml (66) Human Breast milk 321.2 pg/ml (68) Blister 1.8–2.1 pg/blister (69)
**Ortho-Benzo-Resolvin E2** **(** *o*-BZ-RvE2)	(*S*,*E*)-5-hydroxy-8-(2-((*R*,*E*)-3-hydroxypent-1-en-1-yl)phenyl)oct-6-enoic acid	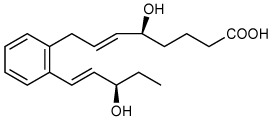	Stops PMN migration (73).	Peritonitis (73).	
**18R-Resolvin E3** (18R-RvE3)	17*R*,18*R*-dihydroxy-5*Z*,8*Z*,11*Z*,13*E*,15*E*- eicosapentaenoic acid	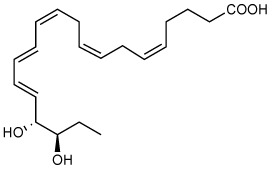	Stops PMN migration (15). Reduces IL-23 and IL-17 (74). BLT1R antagonist (74).	Peritonitis (15), lung inflammation (74), depression (75), and premature birth (76).	SARS-CoV-2 infection (77) Plasma 1.2–1.3 pg/ml (63) Plasma of Arthritis 13.9. pg/ml (64) Arthritis Synovial fluid 95.7 pg/ml (64) Plasma of Type 2 diabetes mellitus 36.9–50.6 pg/ml (65) Plasma of Peripheral artery disease (OMEGA-PAD II trial) 33-187 pg/ml (66) Cord Blood 106.8 pg/ml (67) Human Breast milk 444.9 pg/ml (68) Blister 5.8–11.7 pg/blister (69) Metabolic syndrome (weight loss program) 96–175 pg/4.5 × 10^6^ PMN (70)
**18S-Resolvin E3** (18S-RvE3)	17*R*,18*S*-dihydroxy-5*Z*,8*Z*,11*Z*,13*E*,15*E*-eicosapentaenoic acid	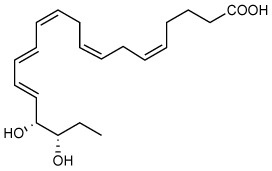	Stops PMN migration (15, 78).	Peritonitis (15, 78).	
**18-deoxy-Resolvin E3** (18-deoxy-RvE3)	17*S*-hydroxy-5*Z*,8*Z*,11*Z*,13*E*,15*E*-eicosapentaenoic acid	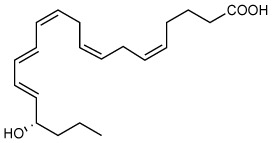	Stop PMN migration (79).	Peritonitis (79).	
**18-hydroxy-eicosapenta-enoic acid** (18-HEPE)	18*S/R*-hydroxy-5*Z*,8*Z*,11*Z*,14*Z*,16*E*-eicosapentaenoic acid	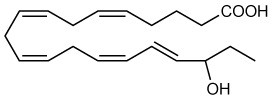	Reduces pro-inflammatory cytokines (16).	Prevents overload-induced maladaptive cardiac remodeling (16) and pulmonary metastasis (80).	

The main actions of RvE4 are to stimulate human macrophage efferocytosis of apoptotic neutrophils and senescent red blood cells, as well as reduce mouse hemorrhagic exudates by increasing efferocytosis and decreasing neutrophil infiltration *in vivo* (17). The ability of RvE4 to limit neutrophil tissue infiltration and to enhance efferocytosis are also functions shared by other members of the E-series resolvins (**Table 1**). For example, RvE1’s pro-resolving actions include stopping neutrophil (12, 14, 37–39) and dendritic cell migration (11, 40), reducing pro-inflammatory cytokines (41), enhancing macrophage phagocytosis and efferocytosis (37, 38), enhancing bacterial clearance (41), modulating T cell responses (42) and regenerating periodontal ligament stem cells (43). RvE1 has unique actions on platelets by inhibiting adenosine diphosphate (ADP)-activated mobilization of P-selectin to minimize platelet aggregation. In addition, RvE1 has potent actions in the nanogram range *in vivo* including: organ protection, clearing infections, and stimulating resolution in, e.g., peritonitis (11, 12, 14, 37, 38), sepsis (45), ischemia-reperfusion injury (46), diabetes (47), colitis (39, 49, 50), lung inflammation (51), obesity (48), atherosclerosis (55), tumor burden (58), dermatitis (59), *Candida albicans* (60), and herpes simplex virus infections (62) as well as in pain (61). In humans, RvE1 has been identified in the plasma of healthy individuals (63), plasma and synovial fluid from arthritic patients (64), plasma of type II diabetes mellitus patients (65), plasma of peripheral artery disease patients (66), cord blood (67), breast milk (68), and blisters induced by UV-killed *E. coli* (69). Recently, RvE1 was also found to be increased in human neutrophils from individuals with metabolic syndrome following weight loss upon stimulation (70). RvE1 analog 18-oxo-resolvin E1 was found to evoke anti-inflammatory actions by reducing neutrophil infiltration and pro-inflammatory cytokine/chemokine production *in vivo* (71). These results demonstrate tissue/organ- and cell type-specific actions of RvE1 in controlling a wide range of diseases where excessive or uncontrolled inflammation is an underlying pathobiology.

In the E-series cascade, RvE2 stops chemoattractant-induced PMN infiltration *in vivo* in murine peritonitis (14) and decreases depression-like behavior in mice (53). RvE2 has also been identified in the plasma of healthy individuals (63), plasma and synovial fluid of arthritis patients correlating with pain reduction in these patients (64), plasma of type II diabetes mellitus patients (65), plasma of peripheral artery disease patients, breast milk (68), and in human blisters induced by UV-killed *E. coli* (69). Of interest, RvE2 analog benzo-resolvin E2 was found to display exceptional potency in the femtomole range in reducing inflammation *in vivo* (73). RvE3 decreases allergic airway inflammation *via* the IL-23/IL-17A pathway (74), reduces depression-like behavior in mice (75), and lowers the incidence of preterm birth in lipopolysaccharide-exposed pregnant mice (76). RvE3 has been identified in the plasma and synovial fluid of arthritic patients (64), plasma of type II diabetes mellitus patients (65), cord blood (67), breast milk (68), and UV-killed *E. coli*-induced human blisters (69). Also, 18-HEPE, a precursor of the E-series resolvins, inhibits macrophage-mediated pro-inflammatory activation of cardiac fibroblasts and prevents overload-induced maladaptive cardiac remodeling *in vivo*, demonstrating its potent actions in cardiac tissue (16). This is further illustrated by the recent discovery of the potent bioactivity of 18-deoxy-resolvin E3 (18-deoxy-RvE3), which stops neutrophil infiltration and resolves peritonitis in mice (79). Of interest, recent results demonstrated that RvE3 is reduced in severe compared to moderate COVID-19 patients (77), highlighting the significance of SPMs in human disease. It has been shown that E-series resolvins are also endogenous anti-depressants (81). These findings from many investigators emphasize the potent structure-based functions and actions of the E-series resolvin members listed in **Table 1**. Further investigations are needed to fully appreciate the structure-activity relationships of RvE4 produced *via* these different biosynthetic pathways discussed above in biological systems.

In summation, the EPA cascade to the E-series bioactive metabolome of resolvins produces several potent mediators within this biosynthesis pathway (**Table 1**) that target diverse cell types relevant to inflammation, resolution, and resolution of vascular inflammation. This EPA precursor cascade is likely to contribute to the clinical impact of omega(ω)-3 supplementation as in recent results from cardiovascular disease patients (82). In recent human randomized trials, omega(ω)-3 supplementation lowered systemic levels of key pro-inflammatory cytokines associated with inflammaging (83). Also, in humans omega(ω)-3 supplements increase RvE1 in serum and plasma of healthy individuals, RvE3 in plasma of patients with peripheral artery disease (66), and circulating RvE1, RvE2, and RvE3 (84). Taken together, our present results establish the complete stereochemistry of the newest member of the E-series resolvins, Resolvin E4 (RvE4: 5*S*,15*S*-dihydroxy-6*E*,8*Z*,11*Z*,13*E*,17*Z*-eicosapentaenoic acid), and confirm its potent bioactions stimulating human macrophage efferocytosis. Moreover, these results provide evidence that synthetic RvE4 can now be used as a standard for LC-MS/MS-based profiling, lipidomics/metabolomic studies as well as help to further define RvE4 specific functions in biological systems and cellular function *in vitro* and *in vivo.*

## Data Availability Statement

The original contributions presented in the study are included in the article/**Supplementary Material**. Further inquiries can be directed to the corresponding author.

## Ethics Statement

Human peripheral blood mononuclear cells (PBMC) were obtained from de-identified healthy human volunteers from the Boston Children’s Hospital Blood Bank under protocol # 1999-P001297 approved by the Partners Human Research Committee.

## Author Contributions

SL carried out the RvE4 bioactions, designed and performed experiments, analyzed the results, and contributed to manuscript and figure preparations. AS performed LC-MS/MS matching, analyzed results, and contributed to manuscript and figure preparations. RN analyzed NMR data and contributed to figure preparation. DF and NW carried out total organic synthesis. NW and MM acquired the NMR data. CS conceived and designed the experiments and contributed to manuscript and figure preparation. All authors contributed to the article and approved the submitted version.

## Funding

This work was supported by the National Institutes of Health (grant nos. P01GM095467 to CS and F32HL142175 to SL).

## Conflict of Interest

The authors declare that the research was conducted in the absence of any commercial or financial relationships that could be construed as a potential conflict of interest.
