# Supplementary figures and images for: A New E-Series Resolvin: RvE4 Stereochemistry and Function in Efferocytosis of Inflammation-Resolution
Source: Front Immunol. 2021 Feb 10;11:631319. doi: 10.3389/fimmu.2020.631319 (PMC7902526; doi:10.3389/fimmu.2020.631319)

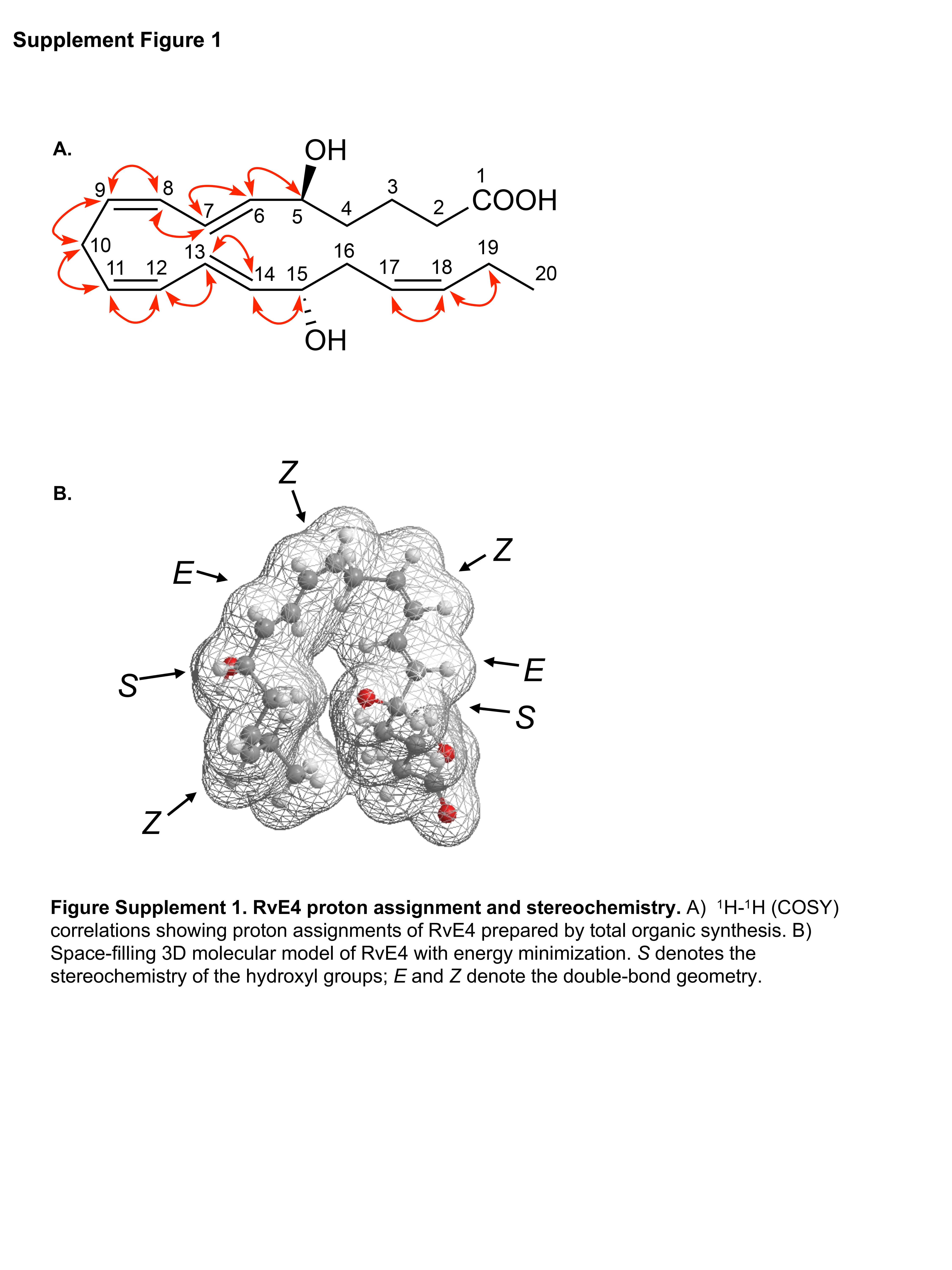

Supplement: Supplementary file 1 [file Image_1.tif]
